# Cloud platform to improve efficiency and coverage of asynchronous multidisciplinary team meetings for patients with digestive tract cancer

**DOI:** 10.3389/fonc.2023.1301781

**Published:** 2024-01-15

**Authors:** Yu Zhang, Jie Li, Min Liao, Yalan Yang, Gang He, Zuhong Zhou, Gang Feng, Feng Gao, Lihua Liu, Xiaojing Xue, Zhongli Liu, Xiaoyan Wang, Qiuling Shi, Xaiobo Du

**Affiliations:** ^1^ Department of Oncology, Mianyang Central Hospital, School of Medicine, University of Electronic Science and Technology, Mianyang, China; ^2^ Information Center, Mianyang Central Hospital, School of Medicine, University of Electronic Science and Technology, Mianyang, China; ^3^ State Key Laboratory of Ultrasound in Medicine and Engineering, School of Public Health, Chongqing Medical University, Chongqing, China

**Keywords:** multidisciplinary team meeting, cloud platform, treatment planning, cancer treatment, digestive tract cancer

## Abstract

**Background:**

Multidisciplinary team (MDT) meetings are the gold standard of cancer treatment. However, the limited participation of multiple medical experts and the low frequency of MDT meetings reduce the efficiency and coverage rate of MDTs. Herein, we retrospectively report the results of an asynchronous MDT based on a cloud platform (cMDT) to improve the efficiency and coverage rate of MDT meetings for digestive tract cancer.

**Methods:**

The participants and cMDT processes associated with digestive tract cancer were discussed using a cloud platform. Software programming and cMDT test runs were subsequently conducted to further improve the software and processing. cMDT for digestive tract cancer was officially launched in June 2019. The doctor response duration, cMDT time, MDT coverage rate, National Comprehensive Cancer Network guidelines compliance rate for patients with stage III rectal cancer, and uniformity rate of medical experts’ opinions were collected.

**Results:**

The final cMDT software and processes used were determined. Among the 7462 digestive tract cancer patients, 3143 (control group) were diagnosed between March 2016 and February 2019, and 4319 (cMDT group) were diagnosed between June 2019 and May 2022. The average number of doctors participating in each cMDT was 3.26 ± 0.88. The average doctor response time was 27.21 ± 20.40 hours, and the average duration of cMDT was 7.68 ± 1.47 min. The coverage rates were 47.85% (1504/3143) and 79.99% (3455/4319) in the control and cMDT groups, respectively. The National Comprehensive Cancer Network guidelines compliance rates for stage III rectal cancer patients were 68.42% and 90.55% in the control and cMDT groups, respectively. The uniformity rate of medical experts’ opinions was 89.75% (3101/3455), and 8.97% (310/3455) of patients needed online discussion through WeChat; only 1.28% (44/3455) of patients needed face-to-face discussion with the cMDT group members.

**Conclusion:**

A cMDT can increase the coverage rate of MDTs and the compliance rate with National Comprehensive Cancer Network guidelines for stage III rectal cancer. The uniformity rate of the medical experts’ opinions was high in the cMDT group, and it reduced contact between medical experts during the COVID-19 pandemic.

## 1 Introduction

Multidisciplinary team (MDT) meetings can provide more reasonable treatment plans for cancer patients, which could prolong their survival and improve their quality of life (1–7). MDT meetings are the gold standard for cancer treatment decisions and are widely used for diagnosing and treating different tumors (8). However, MDT meetings are usually hosted weekly in many hospitals. Different specialists must regularly participate at the same time and place (9–11), which is time-consuming and economically ineffective. Brauer et al. (12) retrospectively analyzed 470 patients with benign and malignant pancreatic and digestive tract diseases, which led to an MDT discussion. They focused on institutional resource utilization for MDT meetings, estimating a cost of 2,035 USD and a total time expenditure of 16.5 hours weekly. Therefore, MDTs are used only in settings that require critical decisions (12). However, MDT meetings are mandatory in the United Kingdom to improve the prognosis of patients with cancer (13). Many cancer patients benefit from MDT meetings; however, balancing MDT efficacy and coverage rate remains challenging.

Internet-based communication has been widely used in the medical care of cancer patients. Telemedicine has been a part of the care of cancer patients during the COVID-19 pandemic (14, 15). Using web conferences to discuss complex or rare cancer cases is reliable and effective for decision-making (16, 17). Virtual multidisciplinary approaches could improve MDT workflow efficiency, shorten the preparation time of MDTs, reduce the meeting time, and yield the same survival results as those in the literature (18, 19). However, few tumor types and cases use web conferences and virtual multidisciplinary meetings; multidisciplinary experts must simultaneously discuss these meetings, which undoubtedly affects the MDT’s coverage rate and efficiency (16–21). Asynchronous communication content has been used between care team members of breast cancer patients, which may improve physicians’ clerical burden and reduce unnecessary interruptions (22).

We propose an asynchronous MDT based on a cloud platform (cMDT) to develop a treatment plan for digestive tract cancer that maximizes the MDT coverage rate for cancer patients and improves MDT efficacy. In the current quality improvement project, we conducted a feasibility study on the implementation of this Internet-based MDT platform, aiming to 1) demonstrate the performance of cMDT in creating a treatment plan for digestive tract cancer, 2) investigate the barriers to implementation, and 3) quantify the burden and compliance with cMDT from the clinicians’ perspective.

## 2 Methods

### 2.1 Study setting

The formation of the cMDT included four steps.

Step one: Establishment of the cMDT. The doctors and administrative staff discussed the following questions: How many groups will be involved in an MDT for digestive tract cancer, and who will be the members of each group? How to perform the cMDT workflow? After four rounds of discussion (one round of discussion every 20 days) from October–December 2018, the participants reached a consensus and proceeded to the next step.

Step two: From January–February 2019, the programmers wrote programs according to the consensus of the cMDT, which was discussed in the first step.

Step three: The cMDT performs test runs and further improves the software and process of the cMDT according to the test run results from March–May 2019.

Step four: The cMDT for digestive tract cancer was officially launched in June 2019.

### 2.2 Data collection

We defined digestive tract cancer patients diagnosed for the first time in our hospital from March 2016 to February 2019 as the control group and those diagnosed from June 2019 to May 2022 as the cMDT group. Patient characteristics, number of doctors participating in each cMDT, doctor response time (the interval between the MDT invitation to the doctor and doctor starting MDT), time of cMDT (total time spent by all MDT participants in a patient), the coverage rate of MDT (the ratio of the number of digestive tract cancer patients who received MDT and the number of digestive tract cancer patients who were diagnosed for the first time), compliance rate with the National Comprehensive Cancer Network (NCCN) guidelines for stage III rectal cancer (the ratio of the number of stage III rectal cancer patients whose treatment plan was consistent with the NCCN guidelines and the number of stage III rectal cancer patients who were diagnosed for the first time) and uniformity rate of medical experts’ opinions (the ratio of the number of digestive tract cancer patients whose treatment opinions were uniform and the number of digestive tract cancer patients who received MDT) were collected.

### 2.3 Statistical analysis

The ages of the patients are presented as the means ± standard deviations. The coverage and compliance rates are expressed as percentages. We used the χ^2^ test or Fisher’s exact test to compare categorical variables between the control and cMDT groups. A *t-*test was used to compare the ages of the patients in the control and cMDT groups. The χ^2^ test was used to compare the coverage rate of MDT between the control and cMDT groups. The χ^2^ test also compared the compliance rate with NCCN guidelines for patients with stage III rectal cancer between the control and cMDT groups. SPSS 24.0 software was used for the statistical analyses.

### 2.4 Ethics approval and consent to participate

This study was approved by the Ethics Committee of Mianyang Central Hospital, Sichuan Province, China (approval number: S-20230340-01). Anonymized patient data from this study were analyzed, and informed consent was not needed.

## 3 Results

### 3.1 Composition of cMDT

**Figure 1:**
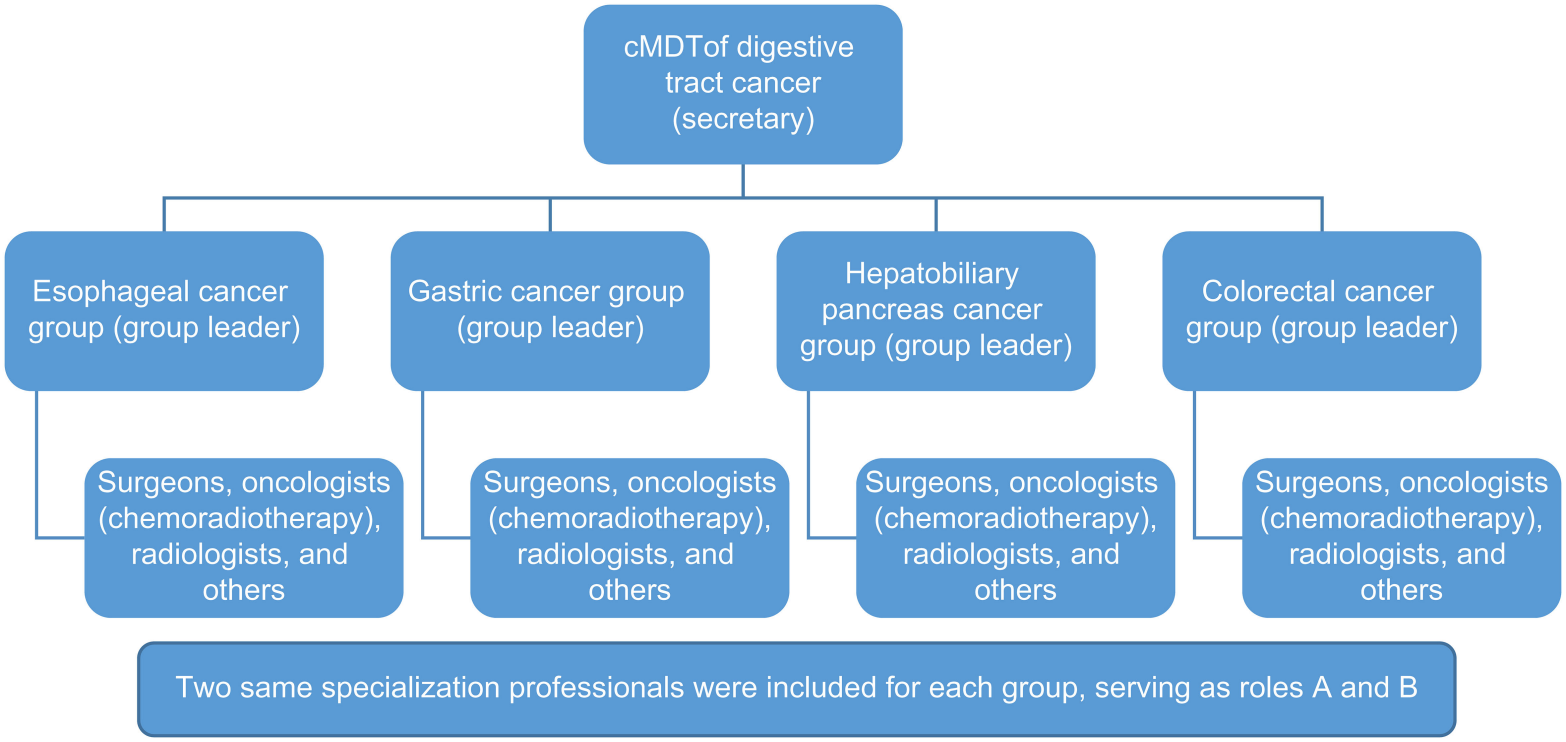
Composition of Cmdt. Digestive tract cancer cMDT was divided into four groups: esophageal cancer, gastric cancer, hepatobiliary pancreas, and colorectal cancer. Two professionals of the same specialization were included in each group, serving as roles A and B.

The cMDT sets up a part-time secretary responsible for the MDT’s operation. The digestive tract cancer cMDT was divided into four groups: esophageal cancer, gastric cancer, hepatobiliary pancreas, and colorectal cancer. Every team has a group leader who hosts offline, face-to-face discussions. The cMDT of each patient included three types of doctors: surgeons, oncologists (chemoradiotherapy), and radiologists. Based on the patient’s condition, other medical experts, including pathologists, nurses, nutritionists, physicians, and intervention doctors, can be invited to participate in the cMDT. Two of the same specialized professionals were included in each group, serving roles A and B. All the cMDT participants had at least 10 years of work experience (**Figure 1**).

### 3.2 cMDT software system

**Figure 2:**
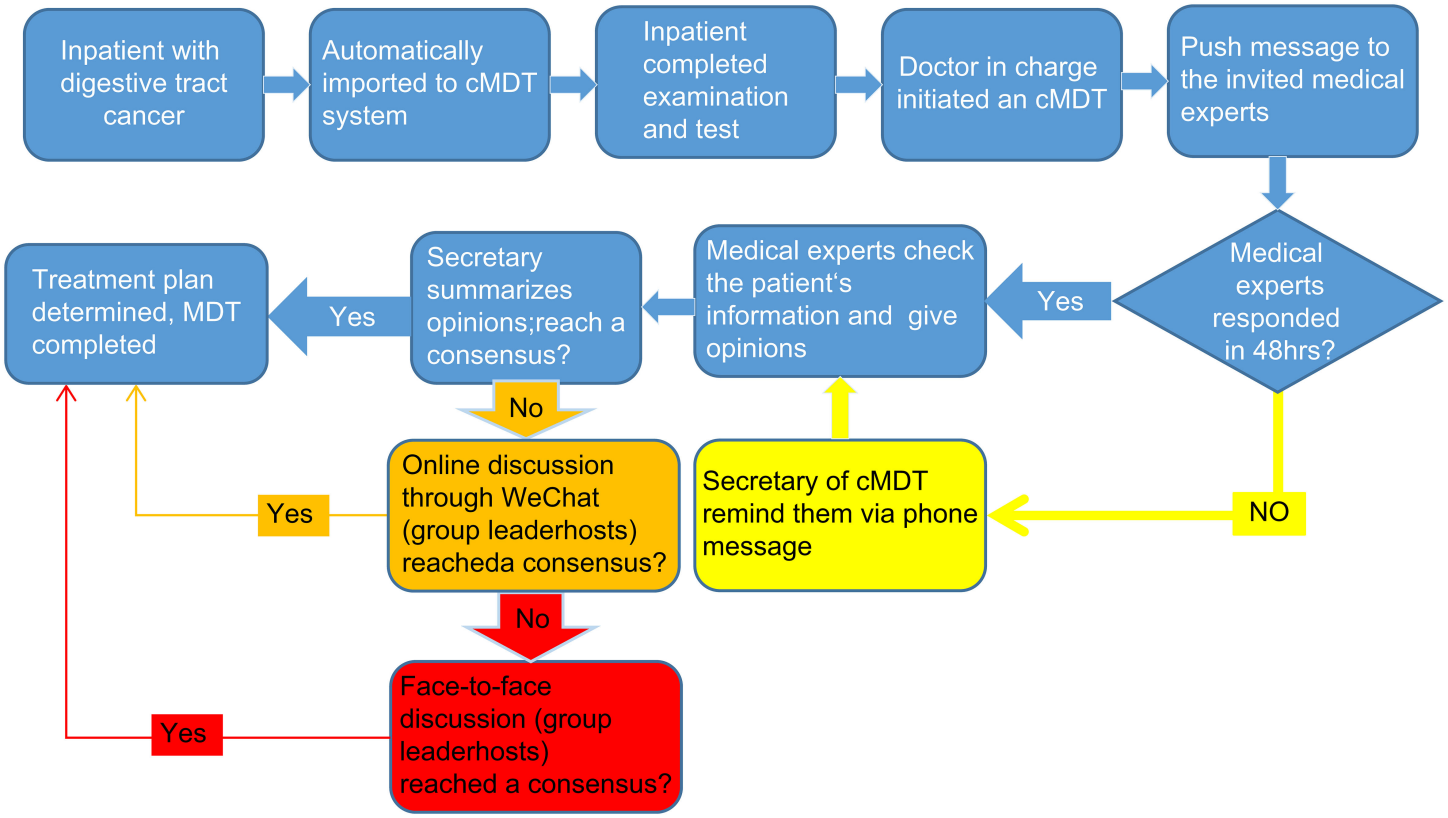
cMDT software system. The cMDT software system on the cloud platform includes four parts: a participant pool, an automatic trigger, patient information, and invited medical experts’ opinions.

The cMDT software system on the cloud platform includes four parts: a participant pool, an automatic trigger, patient information, and invited medical experts’ opinions. The participant pool included all the medical experts on cMDT. This automatic trigger is the first time a patient diagnosed with digestive tract cancer has automatically entered the cMDT system. Patient information included name, sex, age, diagnosis, medical history, and imaging and laboratory examinations. The opinions of the invited medical experts included their opinions and summary opinions (**Figure 2**).

### 3.3 Processes of cMDT

The cMDT system had three test runs. During the test runs, improvements were made to the proposed system. We found that forming only one group that included all medical experts resulted in the invitation information not being pushed accurately; therefore, we divided one group into four groups. When all the invited medical experts provided their opinions, and nobody judged the uniformity of their opinions, we added the secretary’s summary comments. We found that some expert opinions could be reached through simple communication; therefore, we added a WeChat discussion. When medical experts are on vacation or in business, they cannot give their timely opinions; therefore, the number of medical experts in each discipline in each cMDT group increases from one to two, and they are at AB angles to each other. Because more than 50% of the medical experts could not opine within 24 hours, the cMDT secretary reminded them to do so 48 hours after the invitation was sent. Consequently, doctor participation compliance significantly improved, and the number of doctors who needed to be notified manually decreased from 56% to 5%.

**Figure 3:**
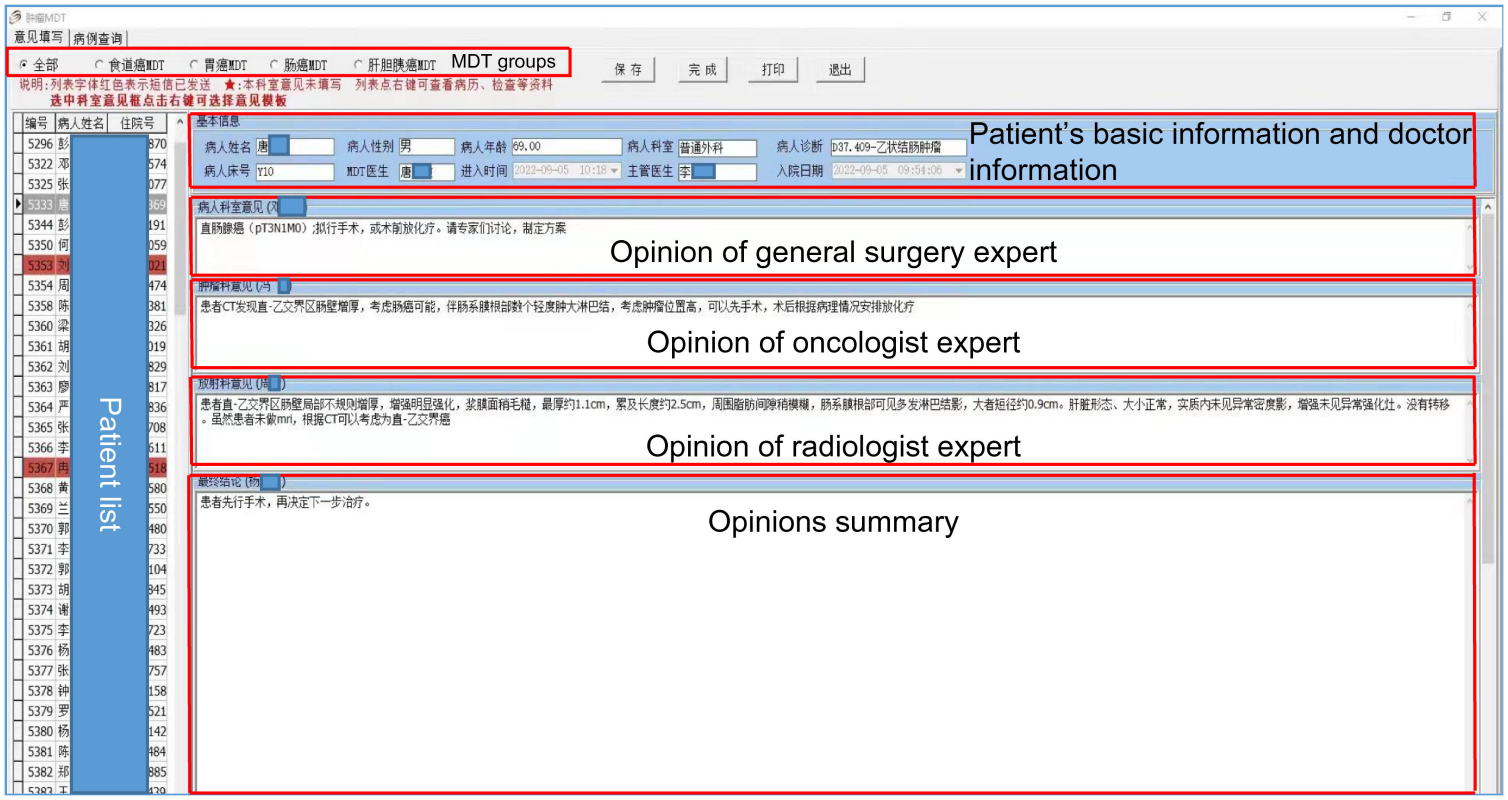
Flowchart of cMDT. When an inpatient is diagnosed with digestive tract cancer, the patient is automatically imported into the cMDT cloud platform by the software system. The doctor in charge initiates a cMDT invitation for other medical experts in the cMDT software system. The system then pushes a message to the mobile phones of invited medical experts (roles A and B). The invited medical experts provided patient treatment opinions. If participants, A and B did not give their opinions 48 hours after the invitation was sent, the secretary of the cMDT reminded them to complete the invitation promptly by phone. After all the invited medical experts provided their opinions, the secretary reviewed and summarized them. If the opinions of all the invited medical experts were consistent, the treatment plan for the patient was determined. If the opinions of all the invited medical experts were inconsistent, the secretary initiated an online discussion through the WeChat group. If the online discussion differed, the team leader organized face-to-face discussions to reach a consensus.

The final process is demonstrated in **Figure 3**. When an inpatient is diagnosed with digestive tract cancer, the patient is automatically imported into the cMDT cloud platform by the software system, which includes the patient’s medical history, examination and test results, and pathological results. After the patient has completed the necessary imaging, laboratory, and pathological tests, the doctor in charge initiates a cMDT invitation for other medical experts in the cMDT software system. The system then pushes a message with the patient’s name, age, diagnosis, department, and bed number to the mobile phones of invited medical experts (roles A and B). The invited medical experts asynchronous checked the cloud platform for the patient’s medical history, image, and laboratory examinations and provided patient treatment opinions. Roles A and B are competitive; only those first entering the system can provide their opinions. If participants, A and B, did not give their opinions 48 hours after the invitation was sent, the secretary of the cMDT reminded them to complete the invitation promptly by phone. After all the invited medical experts provided their opinions, the secretary reviewed and summarized them. If the opinions of all the invited medical experts were consistent, the treatment plan for the patient was determined. If the opinions of all the invited medical experts were inconsistent, the secretary initiated an online discussion through the WeChat group. If the online discussion differed, the team leader organized face-to-face discussions to reach a consensus (**Figure 3**).

After an official operation, the average doctor response time was 27.21 ± 20.40 hours (range between 1 and 98 hours). The average duration of cMDT was 7.68 ± 1.47 minutes (from 5 to 16 minutes), 16.46 ± 3.57 minutes (from 12 to 31 minutes), and 35.52 ± 6.89 minutes (from 25 to 48 minutes) in the cMDT system, WeChat discussion and face-to-face discussion, respectively. According to the cMDT system, 84.98% of the doctors responded at work, 15.02% were off-duty, and more surgeons (25.73%) responded off-duty than other doctors (10.34%). The average number of doctors participating in each cMDT was 3.26 ± 0.88 (range 3 to 8). Among the 11,263 doctors who participated in the cMDT, only 3.18% (358 times) needed to be reminded 48 hours after the message was initiated.

### 3.4 Patient of the cMDT

Among the 7462 patients with digestive tract cancer, 3143 (control group) were diagnosed between March 2016 and February 2019, and 4319 (cMDT group) were diagnosed between June 2019 and May 2022. The patient characteristics are shown in **Table 1**. The coverage rates were 47.8% (1504/3143) and 79.99% (3455/4319) in the control and cMDT groups, respectively. Compliance rates with stage III rectal cancer guidelines were 68.42% and 90.55% in the control and cMDT groups, respectively (**Table 2**). The uniformity rate of medical experts’ opinions was 89.75% (3101/3455), and 8.97% (310/3455) of patients needed online discussion through WeChat; only 1.28% (44/3455) of patients needed face-to-face discussion by multidisciplinary team members in the cMDT group.

**Table 1:** Demographics and characteristics of the control and cMDT groups.

	Patients in Control Group (*n* = 3143)	Patients in cMDT^1^ Group (*n* = 4319)	*P* Value
Age, Mean ± SD^2^, y	66.69 ± 11.27	64.45 ± 10.92	<0.001
Sex Male Female	2418 (76.93) 725 (22.07)	3027 (70.08) 1292 (29.91)	<0.001
Cancer site Esophageal cancer Gastric cancer Pancreatic cancer Cholangiocarcinoma Hepatocellular carcinoma Colon cancer Rectal cancer	1042 (33.15) 778 (24.75) 49 (1.56) 69 (2.20) 236 (7.51) 370 (11.77) 599 (19.06)	1237 (28.64) 1041 (24.10) 111 (2.57) 113 (2.62) 408 (9.45) 572 (13.24) 837 (19.38)	<0.001
Stage Stage I Stage II Stage III Stage IV	346 (11.01) 819 (26.06) 1179 (37.51) 799 (25.42)	548 (14.69) 953 (22.07) 1773 (41.05) 1045 (24.19)	<0.001
MDT^3^ Cancer site Esophageal cancer Gastric cancer Pancreatic cancer Cholangiocarcinoma Hepatocellular carcinoma Colon cancer Rectal cancer Stage Stage I Stage II Stage III Stage IV	1504/3143 (47.85) 511/1042 (49.04) 371/778 (47.69) 28/49 (57.14) 38/69 (55.07) 73/236 (30.93) 173/370 (46.76) 310/599 (51.75) 113/346 (32.66) 381/819 (46.52) 659/1179 (55.90) 351/799 (43.93)	3455/4319 (79.99) 1039/1237 (83.99) 767/1041 (73.68) 81/111 (72.97) 81/113 (71.68) 302/408 (74.02) 456/572 (79.72) 729/837 (87.10) 333/548 (60.77) 827/953 (86.78) 1453/1773 (81.95) 842/1045 (80.57)	<0.001
Esophageal cancer Stage I Stage II Stage III Stage IV	1042 (33.15) 13 (0.41) 306 (9.74) 414 (13.17) 309 (9.83)	1237 (28.64) 18 (0.42) 286 (6.62) 656 (15.19) 277 (6.41)	<0.001
Gastric cancer Stage I Stage II Stage III Stage IV	778 (24.75) 196 (6.24) 110 (3.50) 346 (11.01) 126 (4.01)	1041 (24.10) 283 (6.55) 105 (2.43) 441 (10.21) 212 (4.91)	0.009
Pancreatic cancer Stage I Stage II Stage III Stage IV	49 (1.56) 3 (0.10) 6 (0.19) 18 (0.57) 22 (0.07)	111 (2.57) 8 (0.19) 12 (0.28) 32 (0.74) 59 (1.37)	0.74
Cholangiocarcinoma Stage I Stage II Stage III Stage IV	69 (2.20) 9 (0.29) 12 (0.38) 33 (1.05) 15 (0.48)	113 (2.62) 19 (0.44) 16 (0.37) 46 (1.07) 32 (0.74)	0.59
Hepatocellular carcinoma Stage I Stage II Stage III Stage IV	236 (7.51) 20 (0.064) 56 (1.78) 88 (2.80) 72 (2.29)	408 (9.45) 62 (1.44) 86 (1.99) 132 (3.06) 128 (2.96)	0.08
Colon cancer Stage I Stage II Stage III Stage IV	370 (11.77) 45 (1.43) 151 (4.80) 90 (2.86) 84 (2.67)	572 (13.24) 40 (0.93) 246 (5.70) 159 (3.68) 127 (2.94)	0.05
Rectal cancer Stage I Stage II Stage III Stage IV	599 (19.06) 60 (1.91) 178 (5.66) 190 (6.05) 171 (5.44)	837 (19.38) 118 (2.73) 202 (4.68) 307 (7.11) 210 (4.86)	0.005

^1^Cloud platform-based multidisciplinary term (cMDT).

^2^Standard deviation (SD).

^3^Multidisciplinary term (MDT).

**Table 2:** Compliance rates with guidelines for stage III rectal cancer.

	Control Group (*n* = 190)				cMDT^1^ Group (*n* = 307)				*P* Value
	Guideline^2^ (*n*)	Real-world^3^ (*n*)		Compliance Rate (*%)*	Guideline^2^ (*n*)		Real-world^3^(*n*)	Compliance Rate (*%)*	
Total	190	130		68.42	307		278	90.55	0.05
Neoadjuvant Treatment	126	86		68.25	276		266	96.37	0.04
Surgical Treatment	190	187		98.42	370		366	98.92	0.97
Adjuvant Treatment	187	170		90.90	366		351	95.90	0.68

^1^ Cloud platform-based multidisciplinary term (cMDT).

^2^ Treatment plan recommended by NCCN guideline.

^3^ Treatment plan actually implemented to the patient.

## 4 Discussion

In this quality improvement project, we developed and implemented a web-based MDT in oncology for digestive tract cancer patients. Using a cloud platform on which multidisciplinary professionals can conveniently present their opinions, the modified MDT enables most patients in busy oncological practices to be covered by a standardized and individualized decision-making procedure. For patients whose medical conditions require further discussion, the platform also provides a mechanism for the traditional MDT to reach a consensus on medical decisions. The significantly increased coverage rate of asynchronous cMDT and compliance with clinical practice guidelines demonstrated the benefit of the modified mode of MDT, as it improved the efficiency and effectiveness of patient care.

The UK Department of Health defines an MDT as “a group of people from different healthcare disciplines that meet at a given time (whether physically in one place or by video or teleconferencing) to discuss a given patient, and who are each able to contribute independently to the diagnostic and treatment decisions about the patient” (23). Due to the simultaneous participation of multidisciplinary experts, improving the effectiveness and efficiency of these methods is challenging. Previous studies have shown that the average length of patient discussions is 2–3 minutes (24, 25). Time pressure and excessive caseload affect the quality of MDT decisions (24, 26, 27).A survey based on 1269 MDT members showed that streamlined discussions enhance efficiency and ensure high-quality discussion of complex cases. However, there is also a lack of consensus about the methods by which streamlining can be achieved (28). Another study showed that streamlining the MDT creates additional time within the meeting to discuss more complex clinical cases while allowing all members of the team an opportunity to discuss all patients if needed (29). Another study supported tumor-specific guidance for streamlined MDT discussions (30, 31). This study used the cMDT software system and the cloud platform for asynchronous MDT because experts gave their opinions at different places and times. After digestive tract cancer patients (excluding emergency surgical patients) are automatically imported into the cMDT system, the doctor in charge can advise on radiotherapy, chemotherapy, and surgery only after the patient has completed the MDT. Those who were included in the expert pool had 10 years of work experience to ensure professional opinions from each medical expert. A secretary reminder system and mutual replacement of roles A and B were arranged to ensure the timely implementation of the cMDT. This ensures that the patient’s diagnosis or treatment plan is reasonable. The use of the cMDT system is feasible, because of the limited manual reminders, and the fact that few doctors need assistance with the operation.

The cMDT software system improved the coverage rate of MDT for digestive tract cancer, and the uniformity rate of the medical experts’ opinions was high. Previous studies (32–34) have shown that a low compliance rate with the NCCN guidelines leads to a worse prognosis in cancer patients. An MDT can increase the compliance rate with NCCN guidelines. We chose the compliance rate with the NCCN guidelines for stage III rectal cancer patients as an observation indicator because the treatments included neoadjuvant, surgical, and adjuvant treatments. The use of a cMDT increased the compliance rate with the NCCN guidelines for treating stage III rectal cancer, especially for neoadjuvant treatment; moreover, the use of a cMDT has improved the efficiency of MDT therapy (35, 36). Artificial intelligence (AI) clinical decision support systems (CDSSs) have also been used in MDT for breast cancer, and treatment concordance between the AI CDSS Watson for Oncology (WFO) and a multidisciplinary tumor board occurred in 93% of breast cancer patients. These results suggest that WFO offers an AI computing methodology that may be an effective decision support tool in cancer therapy (27). AI cloud computing is being embedded correctly into infrastructure to help automate routine processes and streamline workloads. Computer-aided diagnosis (CAD) is considered a way to reduce heavy workloads and provide a second opinion to radiologists, as it aids identification and classification of pulmonary nodules as malignant or benign and clarifies the stage of lung cancer (37, 38). AI-based *in vitro* diagnostics have been used in disease detection and disease severity assessment for cardiovascular diseases, COVID-19, and oral cancer (39). The integration of AI in radiotherapy not only autocontours the gross target volume and normal tissue but also plays a role in online adaptive radiotherapy, which saves considerable time for radiation oncologists and physicists and holds the potential for more personalized and efficient cancer care (40–43). Virtual tumor boards were piloted for breast oncology and neuro-oncology, with an optimistic capacity for helping clinicians care for patients with complex needs and address barriers (44). Digital technology could help individuals better connect among the members of multidisciplinary teams. WeChat, QQ, Whatchat, etc. (APPs) with a group chat function can allow members of multiple disciplines to discuss simultaneously or asynchronously in the group through voice or text (45, 46). Additionally, some video conferencing software (Tencent Meeting, ZOOM, Teams, etc.) allows team members of multiple disciplines to have online discussions simultaneously (47, 48). In this study, medical experts still held opinions, but at different times and places, unlike classic MDTs. Doctors can freely arrange their MDT. Because surgeons operate on patients during work time, more surgeons respond off-duty. This approach can avoid the delay caused by waiting for all MDT members to arrive and can save time from the medical department to the MDT location, saving doctors time.

cMDT reduces the MDT preparation time, saving physicians’ time. The progress of MDT is time-consuming and cumbersome; for example, Stahl (49) reported that oncologists took as long as 2 hours to prepare a complex case for review in nearly 47% of health systems. Other specialties, such as radiologists and pathologists, may spend up to 6 hours preparing diagnostic images for a single MDT meeting (34). Digital tumor board solutions have been used to reduce the overall preparation time of MDT for breast cancer, digestive tract cancer, and ear, nose, and throat cancer (50–52). In the last few years, technological developments in the surgical field have been rapid and are continuously evolving. One of the most revolutionizing breakthroughs was the introduction of the internet of things (IoT) concept within surgical practice (53). IoT technology has been used in laparoscopic surgery and can aid in intraoperative, real-time decision-making (54, 55). The IoT is also used for remote monitoring of surgical patients, as it allows doctors and nurses to remotely understand the postoperative condition of patients and provide personalized interventions in a timely manner (56, 57). In this study, almost no patient data needed to be prepared by doctors, except for a few pathological data points. All patient information including data from electronic medical records, laboratory information systems, picture archiving, communication systems, and digital pathology systems, was automatically imported into the cMDT system. The invited medical experts could view all patient information in the cMDT system.

cMDT reduced contact between medical experts during the COVID-19 pandemic. This study began in October 2018, and the pandemic began in December 2019. This study improved MDT coverage and efficiency and objectively reduced contact between medical experts during the pandemic. Restricting movement and gatherings have played a role in reducing COVID-19 transmission rates (58) and have changed the form of MDT. The survey results demonstrated a 63% decrease in the number of MDTs continuing with face-to-face meetings, with the majority making changes, including limiting attendees, social distancing, the use of face masks, and the use of virtual software. There was a decrease in the number of patients discussed, and the quality of the discussion was also limited (59). During the COVID-19 pandemic, virtual multidisciplinary team meetings were held for cancer patients (36, 60, 61). cMDT reduced contact between medical experts and ensured quality, providing a new idea for cancer MDT during respiratory infectious disease pandemics.

This study has several limitations. First, this was a single-center study and not a randomized controlled study, with some results compared to previous data. Second, there was no long-term follow-up data, such as progression-free survival and overall survival data for these patients, which indicates that the effect of cMDT needs to be clarified. Third, cMDT reduces academic communication between doctors of different specialties, especially young doctors (because all cMDT participants must have more than 10 years of work experience), which is not conducive to the growth of young doctors. Engaging young doctors in WeChat discussions or face-to-face discussions may compensate for this disadvantage.

## 5 Conclusion

An asynchronous cMDT based on a cloud platform can increase the MDT coverage rate and guideline compliance rate for patients with stage III rectal cancer, thereby saving doctors time. The uniformity rate in the medical experts’ opinions was high of the cMDT group. In addition, it reduced contact between medical experts during the COVID-19 pandemic.

## Data availability statement

The original contributions presented in the study are included in the article/supplementary material. Further inquiries can be directed to the corresponding authors.

## Ethics statement

The studies involving humans were approved by Ethics Committee of Mianyang Central Hospital. The studies were conducted in accordance with the local legislation and institutional requirements. Written informed consent for participation was not required from the participants or the participants’ legal guardians/next of kin because Anonymized patient data from this study were analyzed, and obtaining informed consent from patients was not required.

## Author contributions

YZ: Data curation, Investigation, Methodology, Project administration, Writing – original draft. JL: Data curation, Investigation, Methodology, Project administration, Writing – original draft. ML: Methodology, Software, Writing – original draft. YY: Data curation, Methodology, Project administration, Writing – original draft. GH: Methodology, Software, Writing – original draft. ZZ: Software, Supervision, Writing – original draft. GF: Project administration, Supervision, Writing – original draft. FG: Data curation, Investigation, Supervision, Writing – original draft. LL: Project administration, Supervision, Writing – original draft. XX: Investigation, Supervision, Writing – original draft. ZL: Investigation, Supervision, Writing – original draft. XW: Investigation, Supervision, Writing – original draft. QS: Conceptualization, Formal analysis, Writing – review & editing. XD: Conceptualization, Formal analysis, Project administration, Supervision, Writing – review & editing.
